# Elucidating the effect of iron acquisition systems in *Klebsiella pneumoniae* on susceptibility to the novel siderophore-cephalosporin cefiderocol

**DOI:** 10.1371/journal.pone.0277946

**Published:** 2022-12-29

**Authors:** Lana Daoud, Farah Al-Marzooq, Carole Ayoub Moubareck, Akela Ghazawi, Timothy Collyns

**Affiliations:** 1 Department of Medical Microbiology and Immunology, College of Medicine and Health Sciences, United Arab Emirates University, Al Ain, United Arab Emirates; 2 College of Natural and Health Sciences, Zayed University, Dubai, United Arab Emirates; 3 Tawam Hospital, Al Ain, United Arab Emirates; Nitte University, INDIA

## Abstract

**Background:**

Cefiderocol (CFDC) is a novel siderophore-cephalosporin, effective against multidrug-resistant Gram-negative bacteria. As it has a siderophore side chain, it can utilize iron acquisition systems for penetration of the bacterial outer membrane. We aimed to elucidate the role of siderophores and iron uptake receptors in defining *Klebsiella pneumoniae* susceptibility to CFDC.

**Methods:**

Initially, 103 *K*. *pneumoniae* strains were characterized for susceptibility to different antibiotics including CFDC. CFDC minimum inhibitory concentrations (MIC) were determined in iron-depleted and iron-enriched conditions. Iron uptake genes including siderophores, their receptors, ferric citrate (*fecA*) and iron uptake (*kfu*) receptors were detected by PCR in all the strains. For 10 selected strains, gene expression was tested in iron-depleted media with or without CFDC treatment and compared to expression in iron-enriched conditions.

**Results:**

CFDC exhibited 96.1% susceptibility, being superior to all the other antibiotics (MIC_50_: 0.5 and MIC_90_: 4 μg/ml). Only three strains (2.9%) were intermediately susceptible and a pandrug resistant strain (0.97%) was resistant to CFDC (MIC: 8 and 256 μg/ml, respectively). The presence of *kfu* and *fecA* had a significant impact on CFDC MIC, especially when co-produced, and if coupled with yersiniabactin receptor (*fyuA*). CFDC MICs were negatively correlated with enterobactin receptor (*fepA*) expression and positively correlated with expression of *kfu* and *fecA*. Thus, *fepA* was associated with increased susceptibility to CFDC, while *kfu* and *fecA* were associated with reduced susceptibility to CFDC. CFDC MICs increased significantly in iron-enriched media, with reduced expression of siderophore receptors, hence, causing less drug uptake.

**Conclusion:**

Iron acquisition systems have a significant impact on CFDC activity, and their altered expression is a factor leading to reduced susceptibility. Iron concentration is also a major player affecting CFDC susceptibility; therefore, it is essential to explore possible ways to improve the drug activity to facilitate its use to treat infections in iron-rich sites.

## Introduction

*Klebsiella pneumoniae* is an important Gram-negative bacterial pathogen that represents a public health threat, coupled with the spread of antimicrobial resistance (AMR) across the globe. There is a continual rise in mortalities related to ineffective treatment as bacteria develop resistance mechanisms against novel antibiotics [1]. Cefiderocol (CFDC) is a novel antibiotic produced to treat multidrug resistant (MDR) and carbapenem resistance (CR) *Enterobacteriaceae*, including *K*. *pneumoniae* [2]. The unique characteristic of CFDC is its chemical structure, as it is considered as the first cephalosporin combined with a siderophore [3]. Natural siderophores are iron-chelating compounds secreted by bacteria to acquire and transport iron across the cell membrane. CFDC has a catechol side chain allowing it to act like a siderophore, by binding to ferric iron (Fe^3+^) and using the iron transport systems found on the outer membrane (OM) of Gram-negative bacteria (GNB) to enter the bacterial cells using trojan-horse approach [4]. CFDC-iron complex can pass into the cell through ferric iron receptors, siderophores receptors as well as passively diffuse through the OM and reach the periplasmic space where iron detaches from the antibiotic [5]. This is especially important because it allows CFDC to be present in high concentrations in the periplasmic space of GNB. CFDC then binds to penicillin-binding proteins, to inhibit peptidoglycan synthesis leading to bacterial death [6].

Iron is a fundamental microelement for bacteria, used as a cofactor with many enzymes involved in vital cellular processes, such as electron transport and cellular respiration [7]. It is usually depleted at the site of infection, as part of host immune response to microbial infections [8]. This phenomenon is known as nutritional immunity where the body uptakes trace minerals to limit the growth of invading pathogens, hence low iron levels are present at the site of infection [9]. Another form of immune response is to block the uptake of siderophores into the bacterial cells by producing molecules like lipocalin-2 which can bind to siderophores preventing them from providing the bacteria with iron, leading to bacteriostatic effect *in vitro* [9]. On the other hand, patients with elevated iron levels due to hemochromatosis or frequent blood transfusions are found to be more susceptible to systemic bacterial infections [10]. It is important to note that there are sites in the human body with high iron content. The liver, for example, is heavily loaded with iron; and to the best of our knowledge, the level of iron depletion in the liver during bacterial infection is not documented in the literature. It is well known that *K*. *pneumoniae* can cause serious infections in the liver, including pyogenic abscesses triggered by hypervirulent strains [11]. To date, CFDC has been FDA-approved for use to treat urinary tract infections and respiratory tract infections, and it is still unknown if it can be effective in treating other types of infection. It is poorly understood how CFDC can penetrate the bacteria in iron enriched environment. Moreover, CFDC is not approved for the treatment of blood stream infections (BSI). In addition, the clinical trials showed that CFDC exhibited higher mortality rates when used to treat BSI [12].

More recent studies have focused on the importance of iron, its uptake mechanisms, and its relation to the pathogenicity of *K*. *pneumoniae*. Chen et al., 2020 tested bacterial growth under different iron concentrations for *K*. *pneumoniae* strains causing liver abscess compared to others that do not cause liver abscess. They have demonstrated that maximum bacterial growth was observed in iron rich media with the highest tested concentration (50 μM) of iron. Another important finding in their study was the siderophore expression rates. They established that all four siderophores, namely enterobactin, aerobactin, yersiniabactin, and salmochelin were expressed in iron depleted condition more than in iron rich media [11].

Bacteria can also acquire iron through other pathways such as *Klebsiella* ferric iron uptake (*kfu)*, and ferric iron citrate receptors (*fecA)*. *FecA* transports ferric iron (Fe^3+^) across the outer membrane and one study identified that *fecA* system contributed to the virulence of enterobactin (*entB*) mutant *E*. *coli* [13]. Limited data is available on these genes in pathogenic strains of *Klebsiella pneumoniae*. Although CFDC is mostly dependent on the iron content at the site of infection, no study documented the effect of iron concentration on the expression of iron acquisition genes and their effect on CFDC activity in *K*. *pneumoniae*.

To the best of our knowledge, iron uptake genes including siderophore receptors (*fepA*, *fyuA*, *iroN*, and *iutA*), ferric iron uptake (*kfu*), and ferric citrate receptor (*fecA*) were not investigated in relation to the activity of CFDC. Therefore, this study aimed to investigate the effect of these iron acquisition genes on the activity of CFDC on a group of *K*. *pneumoniae* strains form diverse clinical sources. It also aimed to test the expression of these genes in response to treatment with CFDC in iron depleted condition compared to the expression obtained in iron enriched and iron depleted conditions without CFDC treatment.

## Materials and methods

### Strains and growth conditions

A total of 103 non-duplicated *Klebsiella pneumoniae* strains were investigated in this study. The bacterial collection included 91 clinical strains isolated from patients attending to hospitals in the UAE including Tawam Hospital, Al-Ain, Dubai hospital and Rashid Hospital, Dubai. The clinical strains were collected from the microbiology laboratories, without revealing patient identity or medical information, in accordance with the obtained ethical approval from Tawam Human Research Ethics Committee, Al-Ain, UAE (approval number: MF2058-2021-761). *K*. *pneumoniae* strains were isolated from multiple infection sites, including urinary specimens (n = 38), respiratory specimens (n = 29), wound or tissue specimens (n = 12), blood (n = 11) and body fluids (n = 1) as shown in **S1 Table**.

Additional 12 strains were purchased from American Type Culture Collection (ATCC, USA) including strains with special characteristics and known resistance gene profiles (**S2 Table**). All the bacteria were preserved in brain heart infusion broth (MAST, UK) with 20% glycerol and stored at– 80°C. The strains were checked for purity before any experiment.

### Antibiotic susceptibility testing

Disk diffusion test was used for antibiotic susceptibility screening using 14 antibiotics covering seven antibiotic categories. Antibiotic disks obtained from Mast, UK, including amoxicillin/ clavulanic acid (20/10 μg), piperacillin/ tazobactam (100/10 μg), cefiderocol (30 μg), cefotaxime (30 μg), cefoxitin (30 μg), ceftazidime (30 μg), cefepime (30 μg), cefpodoxime (10 μg), gentamicin (10 μg), ciprofloxacin (5 μg), trimethoprim-sulfamethoxazole (1.25/23.75 μg), ertapenem (10 μg) and meropenem (10 μg) were applied on Muller Hinton Agar (Oxoid, UK) using Mast^®^Diskmaster Systems dispenser (Mast, UK). The plates were incubated overnight at 37°C. The experiments were performed and interpreted according to the Clinical and Laboratory Standards Institute (CLSI) guidelines [14].

Phenotypic detection of extended spectrum beta-lactamase (ESBL) production was performed using double disk synergy test with cephalosporins and amoxicillin-clavulanate (AUG) [15]. Modified carbapenem inactivation method (mCIM) was used for the phenotypic detection of carbapenamase production and EDTA-CIM (eCIM) method was used for the differentiation between serine carbapenamases and metallo β-lactamases as recommended by the CLSI [14] and as described before [16].

Determination of minimum inhibitory concentration (MIC) was performed as a quantitative measurement of antimicrobial sensitivity using broth microdilution test. As iron is depleted at sites of infection, iron depleted media was prepared as per the CLSI recommendations [14] in order to determine the MIC for CFDC (obtained from MedChemExpress, USA). Briefly, BBL™ Mueller Hinton II Broth, Cation-Adjusted (Becton Dickinson, USA) was prepared according to manufacturer’s instructions. Every 1 L of sterilized broth was mixed with 100g Chelex 100 Resin (Bio-Rad Laboratories, Inc, USA) and stirred for 2 hours to remove cations. Calcium, magnesium, and zinc prepared to reach a final concentration of 20–25 mg/L, 0–12.5 mg/L, 0.5–1.0 mg/L, respectively, were added to the media. The pH of the broth was checked and adjusted to 7.3 and the media was finally sterilized using 0.22 μm filter. In addition to CFDC, MIC testing was performed to determine susceptibility to meropenem (MEM), ceftazidime (CAZ), cefepime (CPM), and colistin (COL) using Muller Hinton broth (Oxoid, UK). These antibiotics were chosen as they are considered comparator antibiotics to CFDC, as described before [16, 17]. List of the strains used in this study with antibiotic susceptibility profiles is shown in **S1 Table**.

### Time-kill studies

Selected strains (n = 8) were chosen to establish the time at which CFDC can kill *K*. *pneumoniae* over a period of 24 hrs treatment. This was important in order to determine the duration of treatment to be used in the gene expression studies. To conduct the test, CFDC was diluted to the concentration of predetermined MIC for each strain. Concentrations prepared were equivalent to ¼, ½, 1, 2, 4, 8, and 16 X MIC, as well as a growth control (untreated culture) without the addition of any antimicrobial agent [18]. An inoculum of bacteria with a concentration of ~ 5 X 10^5^ CFU/ml was used. The test was done in a 96 well plate which was incubated at 37°C during the experiment. Every 2 hrs; starting from 0, 2, 4, 6, and finally 24 hrs after treatment, an inoculum of 5 μl was taken from each well, serially diluted in 0.85% NaCl and spotted on agar for colony counting [19]. After overnight incubation at 37°C, colonies were counted, and bacterial concentration (CFU/ml) was calculated. Each experiment was conducted in duplicate, then mean and standard deviation of bacterial concentration at every time point for each concentration tested were calculated. A semi-log plot was prepared by calculating the log_10_ of the bacterial concentration at every time point for each concentration tested. Log_10_ coordinate was plotted on the Y axis against time on X axis [19, 20].

### Effect of iron depletion and iron enrichment on CFDC activity

Activity of CFDC was tested in iron depleted and iron enriched media. FeCl_3_ (Sigma Aldrich, USA) stock solution was prepared to a concentration of 0.1 M, then added to the iron depleted media to reach a concentration of 100 μM. The level of ferric iron in the culture media was confirmed using a colorimetric iron assay kit (ab83366; Abcam, UK). MICs were determined in iron enriched media for 24 selected strains and were compared to the MICs in iron depleted conditions (list of selected strains is shown in **S3 Table**).

### Detection of genes encoding siderophores and iron acquisition receptors

DNA was extracted from pure bacterial colonies using boiling lysis method [21]. The mechanism of penetration of CFDC through the OM is described in the literature as passing through the iron transport system [22, 23]. As there are multiple genes and receptors responsible for the uptake of iron, we inspected different genes to identify particular pathways for the uptake of CFDC. Two receptors responsible for iron transport were tested including iron uptake system (*kfu*) and ferric citrate receptor (*fecA*). In addition, as CFDC is siderophore-cephalosporin, PCR was used to detect the genes encoding siderophore and siderophore receptors, including enterobactin (*entB*), and its receptor (*fepA*); aerobactin (*iucA*) and its receptor (*iutA*); salmochelin (*iroB*), and its receptor (*iroN*); yersiniabactin (*irp1*) and its receptor (*fyuA*).

For the amplification of each gene, 5x Hot FIREPol^®^ Ready to Load Master Mix (Solis Biodyne, Estonia) was used with a primer concentration of 0.2 μM for each of the forward and reverse primers [24]. List of primers is shown in Table 1. All amplifications were carried out on a Veriti 96-well thermal cycler (Applied Biosystems, USA). PCR products were detected on agarose gel using electrophoresis. Bacterial strains were then compared for the contents of iron acquisition genes in relation to CFDC MICs.

**Table 1:** List of primer sequences used for PCR amplification of iron acquisition genes.

Gene	Primer Sequence (5’-3’)*	Amplicon size	Reference
**Enterobactin (*entB*)**	F: ATATCCCGGCGAACAAGGTC	138	[11]
	R: CGGCGATATTAGCCACCACT		
**Enterobactin-receptor (*fepA*)**	F: CGACGTCTCGGAGATCATT	94	[25]
	R: GATATCAATCTGGCGGTTGTT		
**Aerobactin (*iucA*)**	F: TTTCCTGCTCATCTGGTCAC	103	[25]
	R: CTGGCAGAAAAAGTTGATGC		
**Aerobactin-receptor (*iutA*)**	F: GGCTGGACATCATGGGAACTGG	282	[26]
	R: CGTCGGGAACGGGTAGAATCG		
**Salmochelin (*iroB*)**	F: ACGACGGCGAACCCATTATT	179	[11]
	R: GACTTCACTGGCGGAATCCA		
**Salmochelin- Receptor (*iroN*)**	F: AATCCGGCAAAGAGACGAACCGCC	553	[26]
	R: GTTCGGGCAACCCCTGCTTTGACTT		
**Yersiniabactin (*irp1*)**	F: CTCAGTGGCAACAACAGTGC	151	[11]
	R: GATGGCGCGGTGAATGTTAC		
**Yersiniabactin-receptor (*fyuA*)**	F: TGATTAACCCCGCGACGGGAA	785	[26]
	R: CGCAGTAGGCACGATGTTGTA		
**Iron uptake gene (*kfu*)**	F: ATAGTAGGCGAGCACCGAGA	520	[27]
	R: AGAACCTTCCTCGCTGAACA		
**Ferric Iron Receptor (*fecA*)**	F: CGGGTATGCGTTTCGAACAT	152	[26]
	R: CGAGCCTTCAGTGTTTGCAT		

^*^ F: forward; R: reverse

### Expression of genes encoding siderophores and iron acquisition receptors

#### Selection of bacterial strains for gene expression analysis and treatment groups

From the 103 strains investigated in this study, a total of 10 strains were chosen for gene expression experiments. Strains were selected to encompass a variety of iron acquisition genes; thus, strains harboring 1–4 siderophores and/or different iron transport system genes (*kfu* and/or *fecA*) were included. The collection also composed of strains selected based on susceptibility to CFDC, including some susceptible strains (n = 6), all the intermediately susceptible strains (n = 3), and the only strain which was resistant to CFDC (n = 1).

Three types of treatment were applied, including growth in iron depleted media, iron enriched media and treatment with CFDC in iron depleted media. To test the effect of CFDC treatment on the expression of siderophores and iron acquisition genes, 2 X MIC of CFDC was used to treat bacterial cultures. The concentration was determined as subinhibitory and not bactericidal when used to treat a culture with density of 0.5 McFarland units (~ 10^8^ CFU/ml) to allow for the expression of genes.

#### RNA extraction and cDNA synthesis

RNA was extracted using Monarch Total RNA Miniprep Kit (New England Biolabs, UK) according to the manufacturer’s recommended protocol for RNA isolation from Gram negative bacteria. Briefly, fresh bacterial cultures (equivalent to 0.5 McFarland units) were prepared. RNA was extracted from 2 ml culture at the baseline (time 0), then after 2, 4 and 6 hours of incubation at 37°C in an orbital shaker. Bacterial suspensions were centrifuged at 14,000 rpm for 10 mins to pellet the cells. Cell lysis was done by enzymatic destruction by suspending the pellet in 200 μl of 3 mg/ml lysozyme (Sigma Aldrich, Germany) and incubation at 37°C for one hour. NanoDrop™ 1000 Spectrophotometer (Thermo Scientific, USA) was used for quantification of RNA and to check its purity. Extracted RNA was immediately stored at– 80°C until use [28]. cDNA was prepared with 1μg of total RNA using FIREScript RT cDNA Synthesis Mix (Solis BioDyne, Estonia) with Oligo (dT) and Random hexamer primers. Reveres transcription reactions were carried out in a Veriti 96-well thermal cycler (Applied Biosystems, USA) as follows: primer annealing at 25°C for 10 mins; reverse transcription at 37°C for 30 mins; enzyme inactivation at 85°C for 5 mins. cDNA was stored at– 20°C until use for real-time PCR.

#### Real-time PCR

Real-time assay was carried out to assess the expression of the genes listed in Table 1, as described before [11, 25–27]. The reaction mixture consisted of 0.2 μM primers, 1 μl of cDNA, and 4 μl of HOT FIREPol^®^ EvaGreen^®^ qPCR Supermix (Solis BioDyne, Estonia), adjusted to 20 μl with molecular biology grade water [29]. Real-time PCR was done using the 7500 Real-Time PCR System (Applied Biosystems, USA). Thermal cycling conditions included an initial denaturation step at 95 for 12 min, followed by 40 cycles of denaturation (95°C-15 sec); annealing (60°C-20 sec) and extension (72°C-30 sec), followed by melt curve analysis. Each sample was run in duplicate.

To analyse the generated data, cycle threshold (Ct) values were used to calculate ΔCt using mean CT values generated for each gene subtracted from mean Ct values of the housekeeping gene of the same sample [30]. The gene *rpoB* was used a housekeeping gene for normalization as described before for *K*. *pneumoniae* [11]. Expression Fold Change (RQ) was calculated to study the difference in the expression across different time points and between different treatments.

Fold change (FC) equation used is:

FC = 2^-ΔΔCt^ where ΔΔCt = ΔCt value of target gene–average ΔCt of control.

ΔCt of control was either for time 0 (bassline expression), or the values for untreated culture grown in iron depleted media at the same time point for the same target gene.

### Statistical analyses

Statistical analyses were performed using IBM SPSS Statistics 26.0 software (SPSS Inc., Chicago, IL, USA). Data were expressed as mean ±SD. Mann-Whitney U test was used for the comparison of two independent groups. Two-tailed Spearman’s test was used to examine the correlation between MICs and gene expression in different groups, and to correlate the expression of various iron acquisition genes. Statistical significance was determined at *p* < 0.05. Graphs were generated using GraphPad Prism^®^ Version 9.4.0 (GraphPad Software, Inc., La Jolla, CA, USA). R Project for Statistical Computing software (R version 4.1.2) was used for dendrogram generation and heatmap construction.

## Results

### Characteristics of the strains and response to CFDC treatment

Based on antibiotic resistance profiles, 31% (n = 32) strains were denoted as multidrug resistant (MDR) due to resistance to at least one antibiotic in ≥3 and <6 antimicrobial categories. In addition, 36.9% (n = 38) strains were found to be extensively drug resistant (XDR) due to resistance to one antibiotic in ≥ 6 antimicrobial categories [31]. One strain (KPN 68) was pandrug resistant, due to non-susceptibility to all tested drugs, including CDFC. ESBL production was detected phenotypically in 20.4% (n = 21) of the strains by presentation of keyhole effect between AUG and ≥1 cephalosporin [15]. Carbapenem non-susceptible strains represent 33% (n = 34) of the strains tested and carbapenemase production was confirmed using mCIM and eCIM tests.

Table 2 presents summarized data of the MICs range, MIC_50_ and MIC_90_ of the antibiotics used on the tested bacterial isolates. A full list of all the strains with results of antibiotic susceptibility testing are shown in **S1 Table**.

**Table 2:** MIC distribution for antibiotics (CFDC, MEM, CAZ, CPM and COL) tested on the bacterial strains.

Antibiotics	MIC (μg/ml)			Number of tested strains
	MIC_50_	MIC_90_	Range	
**CFDC—iron depleted media**	0.5	4	≤0.06–256	103
**CFDC—iron enriched media**	8	128	1–128 *	24
**MEM**	0.0625	128	0.0078–128	103
**CAZ**	32	128	0.06–128	103
**CPM**	16	128	0.0078–128	103
**COL**	0.5	8	0.125–16	103

* CFDC MIC increased significantly (*p* <0.001) when tested in iron enriched media for selected strains (n = 24)

CFDC was highly effective against the tested strains with 96.1% (n = 99) susceptibility, while only three strains (2.9%) were intermediately susceptible and one (0.97%) pandrug resistant strain (KPN 68) resistant to CFDC, with MICs of 8 and 256 μg/ml, respectively. CFDC inhibited 91% (n = 61) of strains resistant to one or both parent antibiotics, CPM and CAZ, and was highly effective on MDR and XDR strains including those resistant to carbapenems and colistin.

MICs for CDFC were almost always lower than cefepime, ceftazidime, meropenem and colistin with mean fold difference ± SD, 44.3±75.5, 51.4±77.3, 14.8±39.2 and 3.2±5.3, respectively. This was true for all the strains except one (KPN 8) which was highly susceptible to CPM, CAZ, and MEM exhibiting extremely low MICs (<0.015, 0.25, and 0.031 μg/ml, respectively); however, it showed a higher MIC to CFDC (2 μg/ml), but still in the susceptible range. This finding attracted our attention to the presence of other factors affecting the activity of the drug, namely, iron transport systems.

As shown in Table 2, CFDC MIC increased significantly in iron enriched media (*p*<0.001). CFDC lost its activity against 14/24 strains when compared to CLSI breakpoint with 2–32 folds increase in the MICs when cultured in iron enriched media. List of strains tested in both iron depleted and iron enriched media with their MIC values is shown in **S3 Table**.

**Fig 1:**
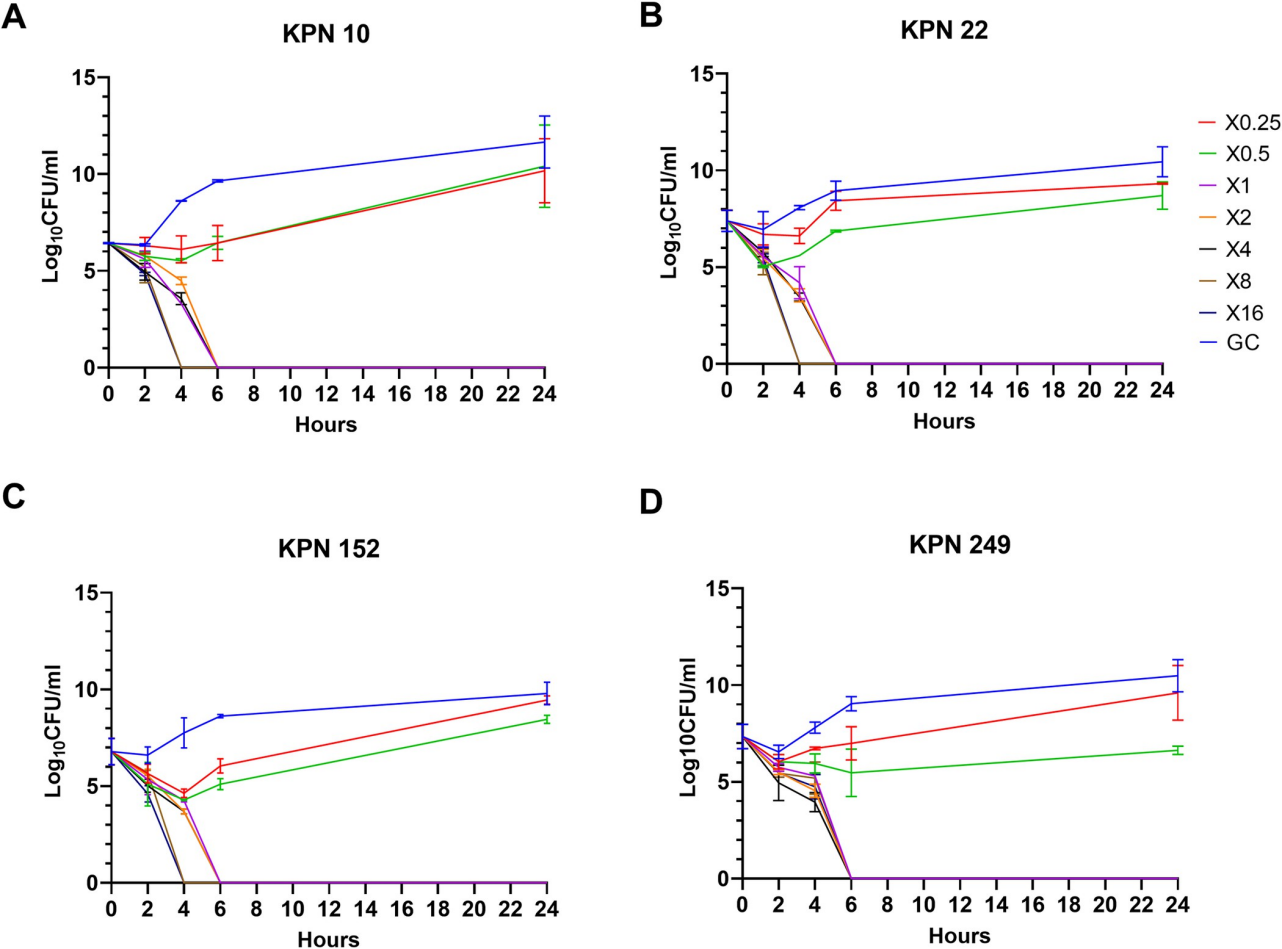
Representative time-kill graphs of four selected strains (A-D) treated with CFDC. Mean of duplicates from two independent experiments ± SD are shown. GC is untreated growth control.

Time-kill assay for CFDC was done to determine the time of killing for use in gene expression studies. Accordingly, bactericidal effect was observed at ≥ 1X MIC at 6 hrs across all tested strains (n = 8). Higher concentrations such as 8 and 16 X MIC had bactericidal effects at 4 hrs time point in 50% of the strains. CFDC showed little effect in the first 2 hrs then, the growth rate started declining rapidly after 4 hrs with concentration ≥ 1X MIC, till complete killing at the 6 hrs time point. Plots of colony count (log_10_ CFU/mL) versus time for representative strains are demonstrated in **Fig 1**.

### Effect of iron acquisition genes on CFDC

**Fig 2:**
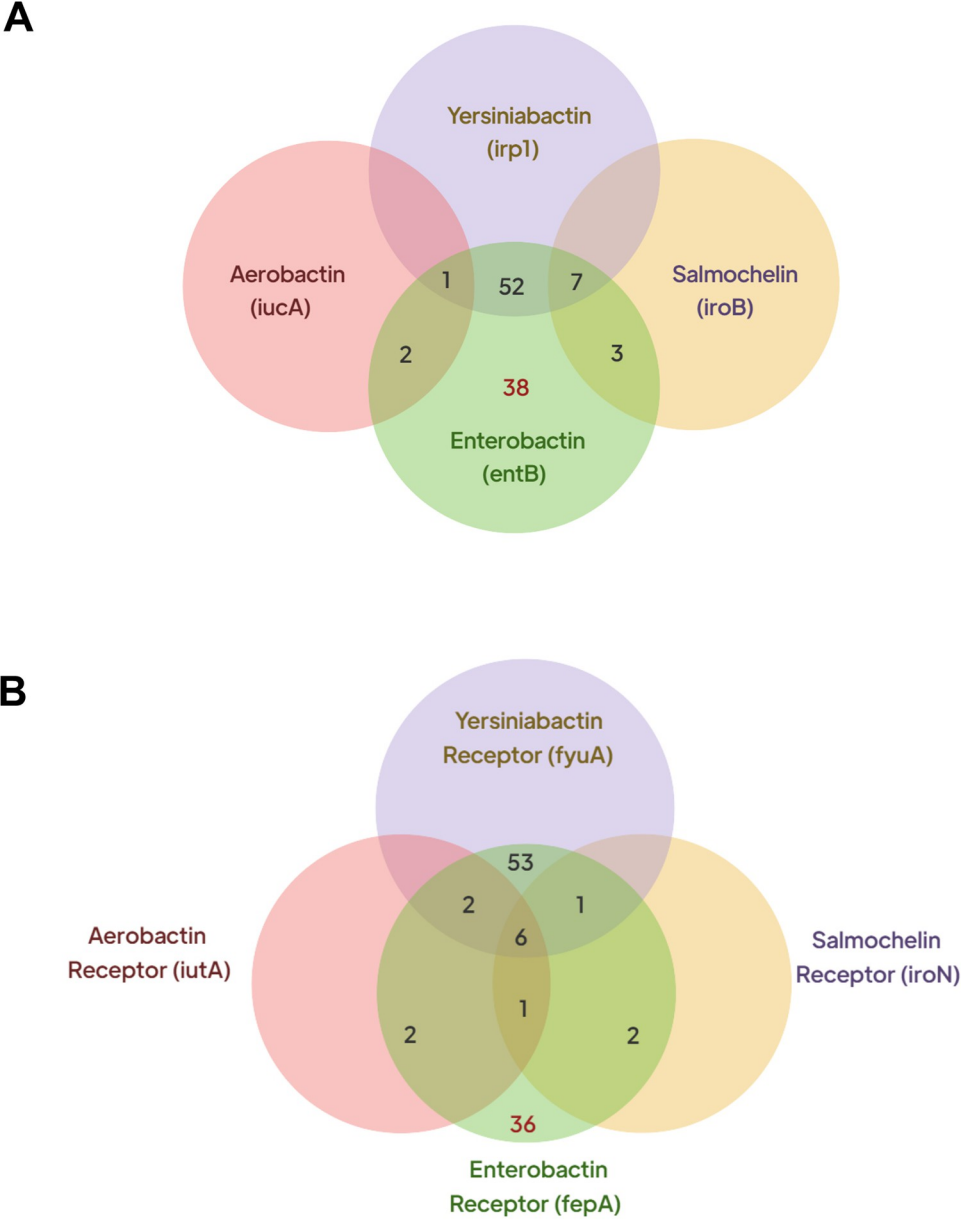
Venn diagrams for siderophore genes and their receptors. Four siderophore genes (A) and their receptors (B) were detected by PCR in 103 *Klebsiella pneumoniae* strains investigated in this study. The numbers shown represent the number of strains containing the genes.

As shown in **Fig 2**, the tested 103 strains contained different combinations of siderophores (Fig 2A) and siderophore receptors (Fig 2B).

As shown in Fig 2A, enterobactin (*entB*) was detected in all 103 strains, and 38 strains (36.9%) produced *entB* only without other siderophores. The co-production of other siderophores in addition to *entB* is shown in the overlapping areas between the labelled sections of the Venn diagram. As shown in Fig 2B, all 103 strains produced the enterobactin receptor (*fepA*), with only 36 strains (34.95%) had this receptor alone, while the rest of the strains carried other receptors accompanied by *fepA*. Although no strain produced the four siderophores, a few strains (n = 6; 5.8%) produced the four receptors.

Table 3 shows the combinations of iron acquisition receptor genes found in the strains, and effects on CFDC MICs.

**Table 3:** Combinations of iron acquisition receptor genes found in the strains and their effect on CFDC MICs.

Receptor genes ^¥^		N (%)	CFDC MIC (μg/ml)		*p* value
			MIC_50_	Range	
**Enterobactin (*fepA*) alone (control group)** ^**#**^		9 (8.7)	0.06	0.02–4	*-*
***fepA* +**	**Yersiniabactin receptor (*fyuA*)**	15 (14.6)	0.125	0.03–2	0.155
	**Ferric citrate receptor (*fecA*)**	17 (16.5)	1	0.06–8 ^‡^	0.013*
	**Iron uptake receptor (*kfu*)**	5 (4.9)	2	0.125–4	0.024*
	***fecA + fyuA***	14 (13.6)	0.5	0.25–2	0.53
	***kfu + fyuA***	19 (18.4)	1	0.03–256 ^‡^	0.028*
	***kfu + fecA***	10 (9.7)	0.5	0.03–2	0.113
	***kfu + fecA + fyuA***	14 (13.6)	1	0.125–8 ^‡^	0.011*

# group of strains harbouring enterobactin receptor (*fepA*) was used as a control group, for comparison to each of the groups with other receptors.

* significant difference

^‡^ intermediately susceptible (MIC = 8 μg/ml), or resistant (MIC = 256 μg/ml)

^¥^ some strains contained other receptors (e.g. *iutA*, and/or *iroN*) and were grouped together for purpose of statistical comparison

As shown in Table 3, the production of either ferric citrate receptor (*fecA*) or iron uptake receptor (*kfu)* with enterobactin receptor (*fepA*) had a significant effect on CFDC MIC. They also caused a significant increase to the MIC of CFDC when they were co-produced with yersiniabactin receptor (*fyuA*). The presence of *kfu* with *fyuA*, even without *fecA*, also caused a significant increase in CFDC MICs in some strains.

### Gene expression

Gene expression (GE) of siderophores, siderophore receptors and iron transport genes was assessed in 10 strains, including CFDC resistant (R; n = 1), intermediately susceptible (I; n = 3), and susceptible (S; n = 6) strains. Table 4 is a list of the strains used in GE and their properties.

**Table 4:** Strains tested for gene expression and their properties including genes encoding iron acquisition systems and CFDC MICs in iron depleted and iron enriched media.

Strains (CFDC susceptibility)	Antibiotic susceptibility profile *	Genes for siderophores	Genes for siderophore receptors	Iron uptake genes	CFDC MIC (μg/ml)		
					Iron depleted media	Iron enriched media	Fold change^$^
**KPN 68 (R)**	PDR	*entB*, *irp1*	*fepA*, *fyuA*	*kfu*	256 ^‡^	>256 ^#^	ND
**CDC AR0066 (I)**	XDR, ESBL, CR	*entB*, *irp1*	*fepA*, *fyuA*	*fecA*, *kfu*	8 ^‡^	256 ^#^	16
**NCTC 13439 (I)**	MDR, ESBL, CR	*entB*	*fepA*	*fecA*	8 ^‡^	256 ^#^	16
**KPN 63 (I)**	ESBL	*entB*, *iroB*	*fepA*, *iroN*	*fecA*	8 ^‡^	32 ^#^	4
**KPN 249 (S)**	XDR, CR	*entB*, *irp1*	*fepA*, *fyuA*	*kfu*	4	128 ^#^	32
**KPN 8 (S)**	SUS	*entB*, *irp1*, *iroB*	*fepA*, *fyuA*, *iutA*	none	2	4	2
**KPN 23 (S)**	XDR, CR	*entB*, *irp1*	*fepA*, *fyuA*	*kfu*	2	8	4
**CDC AR0039 (S)**	XDR, ESBL, CR	*entB*	*fepA*	none	0.5	1	2
**KPN 62 (S)**	MDR	*entB*	*fepA*, *fyuA*	*fecA*, *kfu*	0.5	2	8
**KPN 7 (S)**	SUS	*entB*	*fepA*	none	0.03	<0.06	ND

* MDR: multidrug resistant; CR: carbapenem resistant; XDR: extensively drug resistant; PDR: pandrug resistant; ESBL: extended spectrum beta-lactamase, SUS: susceptible to all antibiotics tested

^‡^ intermediately susceptible (MIC = 8 μg/ml), or resistant (MIC = 256 μg/ml)

^#^ MIC > 8 μg/ml: CFDC resistant based on CLSI breakpoints in iron depleted media

^**$**^ fold increase in CFDC MIC in iron enriched compared to iron depleted media; ND: not determined (MIC was too low, or too high)

**Fig 3:**
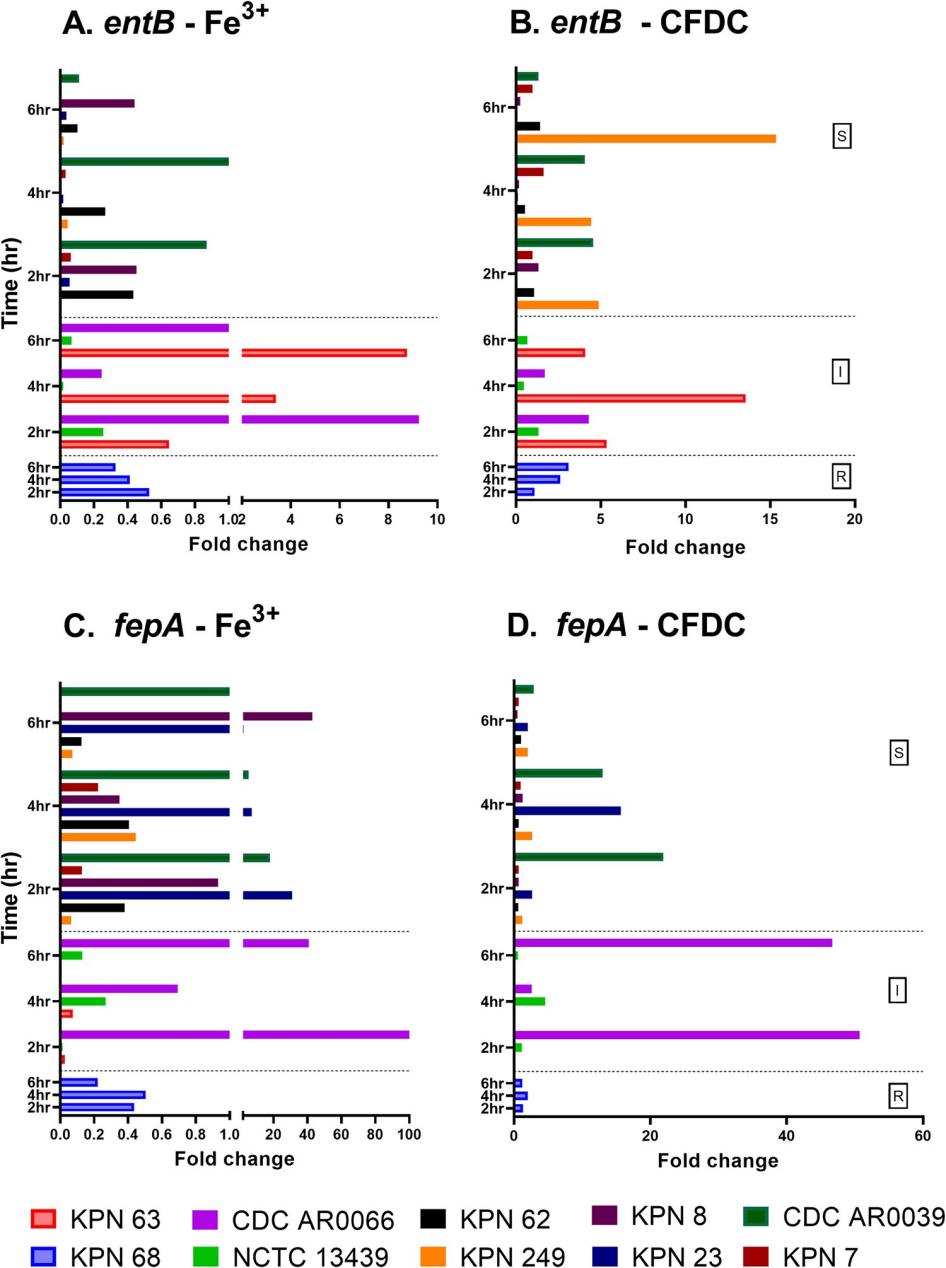
Gene expression of enterobactin (*entB*) and its receptor (*fepA*) in strains overtime in different media. Fold changes of expression were calculated for strains grown in different media by comparing the expression of each gene for bacteria treated with CFDC or grown in iron enriched media to the expression of the same gene at the same time point when the bacteria were grown in iron depleted media.

**Fig 4:**
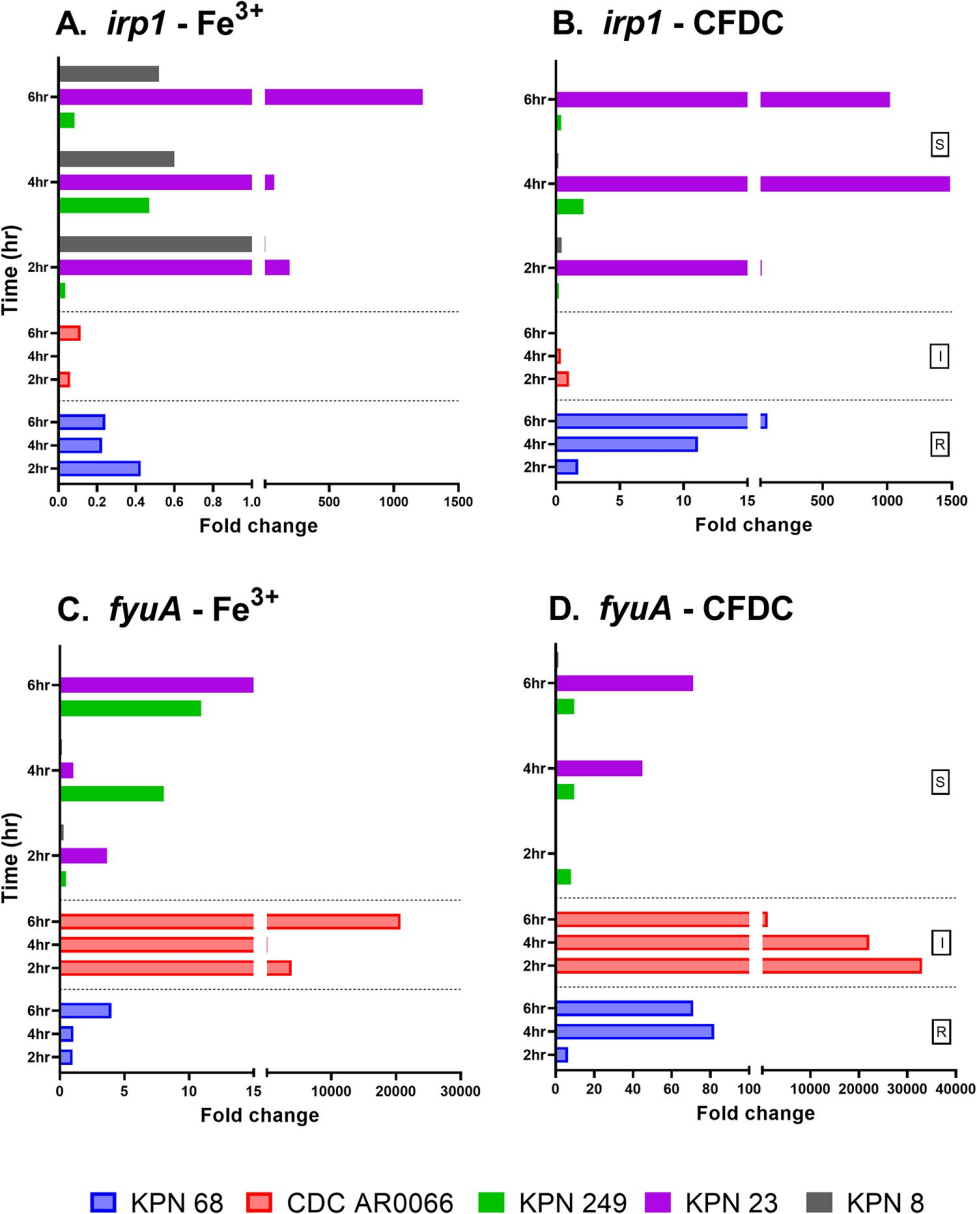
Gene expression of yersiniabactin (*irp1*) and its receptor (*fyuA*) in strains overtime in different media. Fold changes of expression were calculated for strains grown in different media by comparing the expression of each gene for bacteria treated with CFDC or grown in iron enriched media to the expression of the same gene at the same time point when the bacteria were grown in iron depleted media.

**Fig 5:**
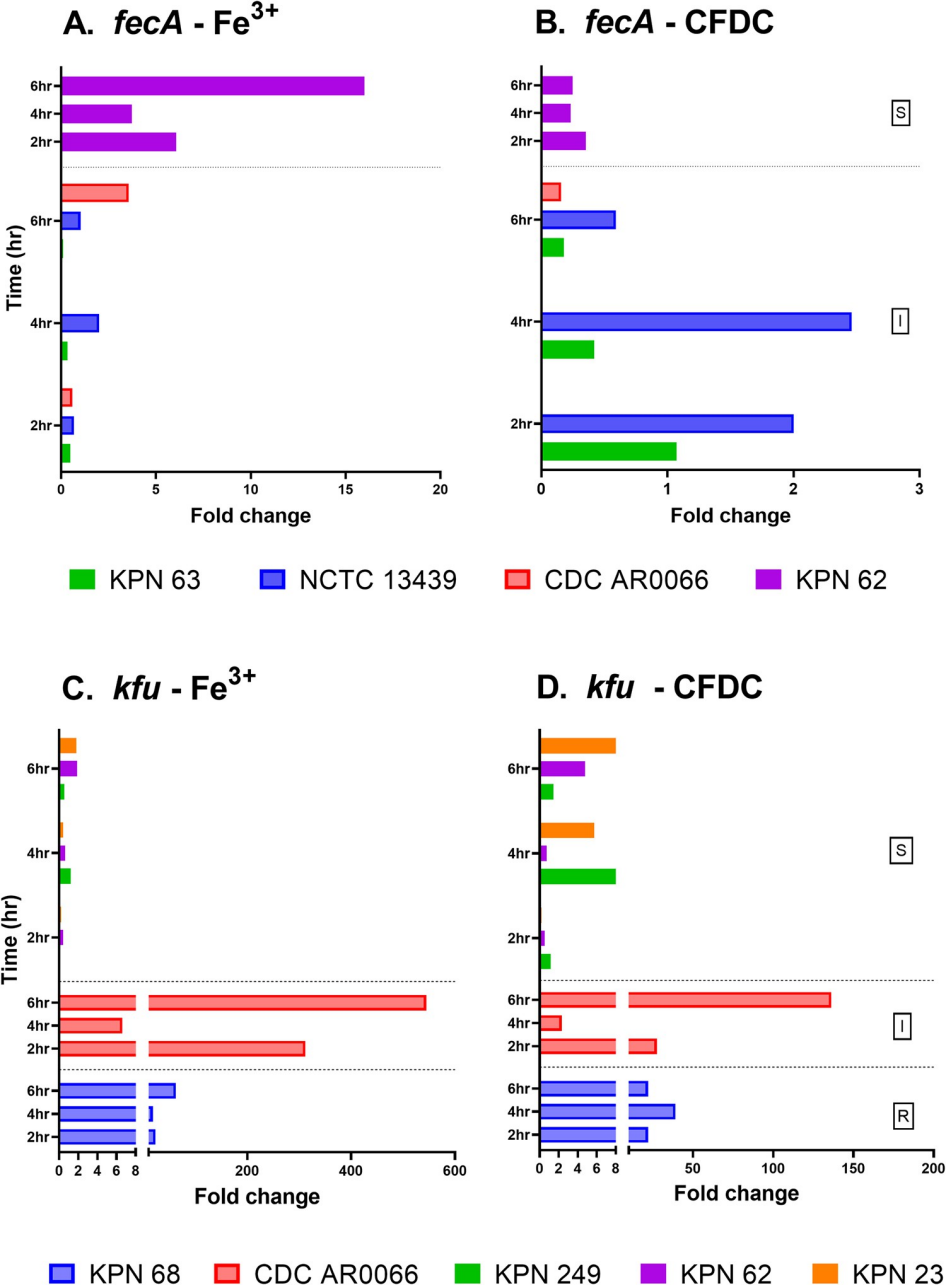
Iron transport gene expression of strains overtime in different media. Fold changes of expression were calculated for strains grown in different media by comparing the expression of each gene for bacteria treated with CFDC or grown in iron enriched media to the expression of the same gene at the same time point when the bacteria were grown in iron depleted media.

**Fig 6:**
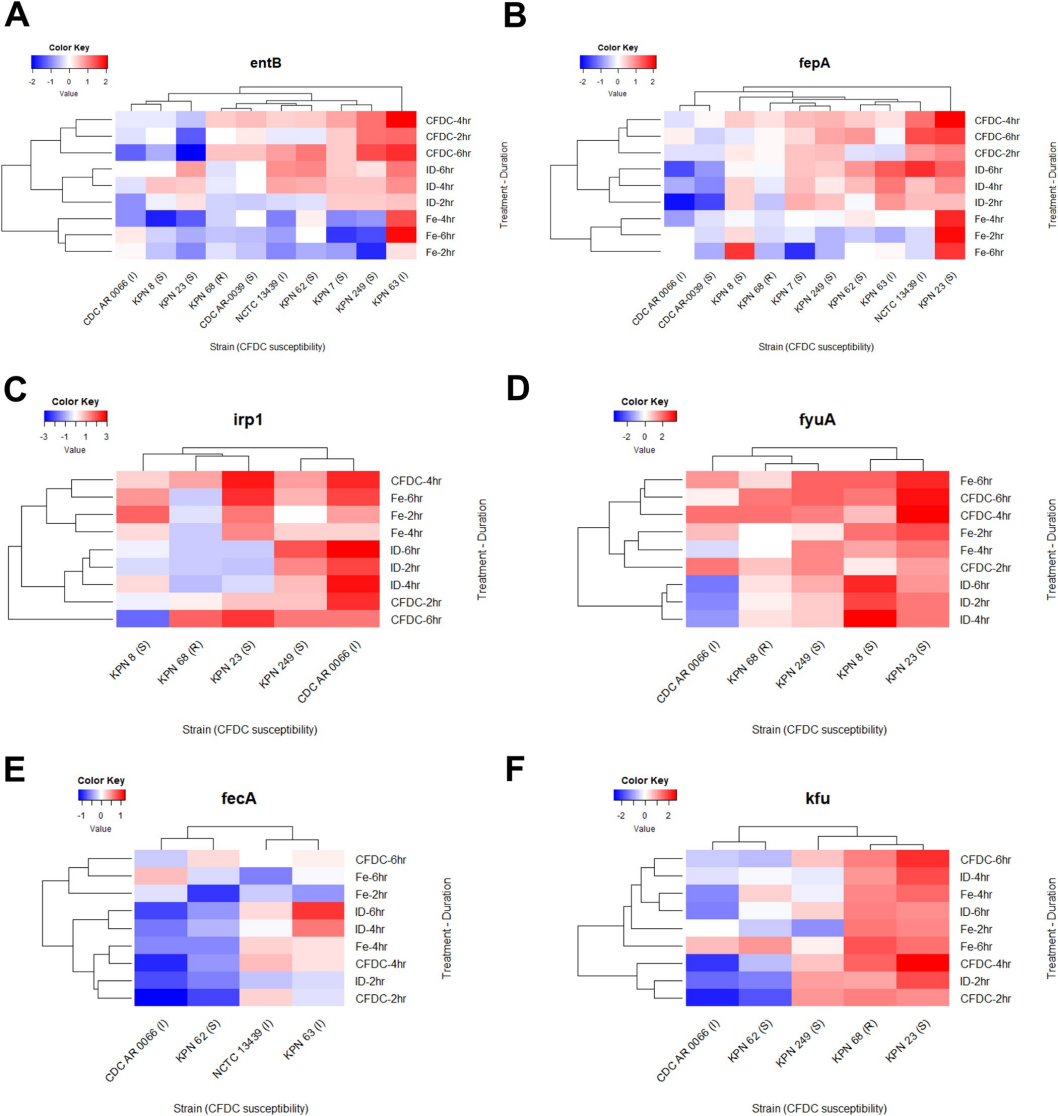
Heatmaps showing the expression of the genes encoding siderophores, their receptors with other iron transport receptors. Expression of siderophore genes including *entB* and *irp1* (A and C), siderophore receptors *fepA* and *fyuA* (B and D), *fecA* ferric iron transport receptor (E) and *kfu* iron uptake receptor (F) are shown. Fold changes of expression were calculated for strains grown in different media by comparing the expression of each gene to the baseline expression for the same gene at 0 hr.

GE in iron enriched media and CFDC treated cultures were compared and normalized to expressions detected in iron depleted media at the same time point of treatment. Folds change in expression are shown in Figs 3–5. Additionally, GE in iron depleted media, iron enriched media and CFDC treated cultures were compared and normalized to expressions detected in time 0 (as baseline expression) of bacteria suspended in iron depleted media. Fold changes in expression of all strains in the three media are represented as heatmaps in Fig 6 to demonstrate the patterns of gene expression.

It was observed that maximum expression of all iron acquisition genes was reached at 4 hrs in some strains and at 6 hrs in the others in different growth conditions (Figs 3–6). As shown in Fig 3, enterobactin (*entB*) and its receptor (*fepA*) were detected in all the 10 strains, while the rest of the siderophore genes and their receptors were expressed in some strains as shown in Figs 4 and 5.

GE of *entB* siderophore was significantly higher in I group than S group in iron enriched media (*p =* 0.023) (Fig 3B). GE of *fepA* was significantly higher than the expression of *entB* when bacteria were cultured in iron depleted media with or without CFDC. As shown in Fig 3C and 3D, *fepA* expression was significantly lower in bacteria grown in iron enriched media than bacteria treated with CFDC (*p* = 0.015). After checking GE patterns in heatmaps for *entB* (Fig 6A) and *fepA* (Fig 6B), both genes were more expressed when bacteria were cultured in iron depleted media with or without CFDC than in iron enriched media. GE of *entB* was highly variable between strains in I group, as CDC AR0066 under expressed the siderophore while KPN 63 over expressed it when cultured in all three media (Fig 6A). In general, *entB* was downregulated in all strains when cultured in iron enriched media except for KPN 63 (I). Also, *fepA* GE was unique in the latter strain, being expressed highly in iron depleted media without CFDC than iron enriched media. Generally, GE of *fepA* was lower when the bacteria were grown in iron enriched media, except for the KPN 23 (S) and KPN 8 (S), whereby the expression did not significantly vary across the different media. Strain KPN 8 was also unique as *entB* gene was downregulated in iron depleted media with or without CFDC. Same was noted for KNP 23 (S) although the expression was higher when grown in iron depleted media without CFDC. The resistant strain (KPN 68) also demonstrated different pattern as both *entB* and *fepA* were less expressed in all media, except with CFDC treatment. Other unique patterns for *fepA* were noted in strains CDC AR0066 (I) and CDC AR0039 (S) which have known outer membrane porin (OMP) mutations, and the gene was downregulated in all the media (Fig 6B), in contrast to all the other bacteria.

As shown in Fig 4, yersinibactin (*irp1*) was highly expressed in all strains except in KPN 8 at the end of CFDC treatment period (6 hrs), and the receptor (*fyuA*) was more expressed than its siderophore (*irp1*) in all media (Fig 4). The expression of *fyuA* was high in all the strains, except CDC AR0066 (I) whereby the expression was low when cultured in iron depleted media without CDFC, while the expression was increased in iron enriched media (*p* = 0.400) as shown in Fig 6D. As for the resistant strain (KPN 68), it also demonstrated a unique pattern for expression of *irp1* and *fyuA*, showing higher expression when treated with CFDC compared to other growth conditions.

Overall, the expression of *fecA* was lower than the other receptors, and the difference was statistically significant in majority of the strains (*p*<0.05). As shown in Fig 5A and 5B, *fecA* receptor was produced in all strains from I group; however, GE was low and non-significantly increased overtime in iron enriched media (*p =* 0.70). The expression of *fecA* in strain KPN 62 (S) was significantly higher than the expression of the I group cultured in iron enriched media (*p =* 0.009). On the contrary, the expression of *fecA* receptor in the S group was more in iron enriched media than in CFDC treated cultures. When checking GE patterns in the heatmap (Fig 6E), overall higher expression was noticed in the I group except for strain CDC AR 0066.

As for *kfu*, GE was significantly higher in R group than S group (*p =* 0.009) in bacteria grown in iron enriched media (Fig 5C). Even in CFDC treated bacteria, GE in R group was significantly higher than S group (*p =* 0.009) as shown in Fig 5D. *Kfu* was overexpressed in KPN 68 (R) and KPN 23 (S), and its expression was consistent when these two strains were cultured in all the media (Fig 6F). The expression of *kfu* gene in S group was lower in the presence of iron, and the expression was significantly lower than the other groups (*p =* 0.009). As seen for *fecA*, strain CDC AR0066 exhibited a unique pattern as *kfu* was under expressed in all the three growth conditions (Fig 6F). It is noteworthy that strains CDC AR 0066 and KPN 62 exhibited similar expression patterns of both *fecA* and *kfu*, which were co-produced in these strains only, but the GE was lower compared to the other strains which expressed either *fecA* or *kfu*, but not both (Fig 6E and 6F). Furthermore, the vast majority of strains producing *fecA* and/or *kfu*, had unique expression patterns, with either overexpression or under expression of siderophores and siderophore receptors (Fig 6A–6D).

### Correlation between GE of different iron acquisition genes

Correlation analysis for GE of iron acquisition genes in iron depleted media revealed a strong positive correlation between the expression of *entB* and siderophore receptors *fepA* and *fyuA* (*p* <0.001 and 0.024, respectively). There was a strong positive correlation between the expression of *irp1* and *fyuA* (*p =* 0.003). Also, there was a strong positive correlation between the expression of *fecA* and *kfu* at 2 and 4 hrs, but the correlation was negative at 6 hrs. In iron enriched cultures, there was a strong positive correlation between *fepA* with *irp1* and *fyuA* (*p =* 0.003 and 0.002, respectively), while there was no correlation with *entB* (*p =* 0.254).

### Correlation of GE of iron acquisition genes with CFDC MICs

Although non-significant, a negative correlation was found between the expression of both *entB* and *fepA* receptor with the MICs of bacteria cultured in all three media at all the time points. Also, there was negative correlation between the expression of *irp1* and *fyuA* receptor and the MIC during growth in iron depleted media and iron enriched media (significant at 6 hrs). On the contrary, there was positive correlation between the expression of *fecA* and the MIC of bacteria cultured in iron depleted media and iron enriched media. Lastly, positive correlation was seen between the expression of *kfu* and the MIC for CFDC treated bacteria at all the time point, and at the end of treatment time (6 hrs) in the other media.

## Discussion

Cefiderocol is the first in class siderophore-cephalosporin that is FDA approved for clinical use as an advanced-generation cephalosporin with a catechol iron-binding moiety [32]. There are reports of resistance to this drug, as well as variability in MICs in clinical strains of GNB [33, 34]. Due to its unique structure and mechanism of action, it is crucial to understand the factors affecting the drug activity. In this study, we investigated 103 *K*. *pneumoniae* strains from diverse clinical sources with various antibiotic susceptibility profiles. In agreement with other reports [14, 19], CFDC was superior to all the other antibiotics, with potent activity against XDR, MDR, CR, and ESBL producing strains, and even on strains resistant to colistin, one of the last-resort antibiotics [35]. However, a few strains were non-susceptible to the drug, including a strain that was pandrug resistant (KPN 68). Furthermore, one strain exhibited unusual profile (KPN 8) as it had lower susceptibility to CFDC than other cephalosporins and carbapenems. Therefore, we tested the expression of iron acquisition genes that might affect the entry of the drug to the bacterial cells in addition to other factors, considering bacterial susceptibility to the drug.

The efficacy of CFDC was first tested in iron depleted media in order to mimic iron depleted conditions *in vivo* [36]. Then, the efficacy of CFDC was tested in iron enriched medium to mimic the increased iron levels in sites with high iron concentrations. Upon testing CFDC in iron enriched media, there was a significant increase in the MIC. This is concerning as it indicates that CFDC will be ineffective when used in iron dense infection sites. Another report documented the loss of activity of CFDC in the presence of high extracellular iron in *Pseudomonas aeruginosa* [23]. The latter study proved that CFDC activity is influenced by the iron concentration in the media, and the investigators suggested that this can be attributed to the increased expression of the iron-regulated outer membrane proteins, but that was not proven experimentally, and no specific iron acquisitions systems were tested. In our study, the activity of CFDC was affected in iron enriched conditions; thus, it was interesting to link the expression of iron acquisition systems to the drug activity. Given that CFDC has a catechol side chain, it can bind iron and penetrate the OM by the iron transport systems. It can be uptaken into bacterial cells as other iron acquisition molecules using receptor mediated pathway [37]. Taking these facts into consideration, we investigated the effects of iron acquisition genes: siderophores and their receptors, iron transport (*kfu*), and ferric citrate receptor (*fecA*) that might be related to CFDC activity. The genes were first detected by PCR, then their expression was tested by real-time PCR. To the best of our knowledge, this is the first study linking the selected siderophore receptors, and iron transporters to CFDC activity.

Based on the results of the time-kill study, CFDC produced bactericidal effect at ≥ 1X MIC after 6 hrs exposure to the drug. Thus, gene expression studies were done with maximum treatment duration of 6 hrs. Our results showed that maximum expression of iron acquisition genes was reached after 4–6 hrs of treatment. Importantly, enterobactin siderophore receptor (*fepA*) expression was highest between 4 and 6 hrs of treatment, which can explain the killing time, considering that drug uptake can be facilitated by this siderophore receptor. As *entB* is the major catechol siderophore in *K*. *pneumoniae* [38], it is possible that its receptor *fepA* can act as a major pathway of CFDC entry to the bacterial cell. In fact, enterobactin (*entB*) and its receptor (*fepA*) were found to be highly produced in *K*. *pneumoniae* [39]. Consistent with these findings, *entB* and *fepA* receptor were detected in 100% of our strain collection. Correlation analysis showed a negative relation between CFDC MICs and GE of *entB* and *fepA* receptors. Although the results were not statistically significant, possibly due to small sample size, they reflect that higher expression of these genes can lead to lower CFDC MICs. It is important to mention that we reported reduced susceptibility to CFDC in strains with downregulated *fepA* as in CDC AR0066 (I) and KPN 68 (R), suggesting a possible role of *fepA* in facilitating the drug uptake. This is also supported by the finding that expression of *entB* and *fepA* was higher in CFDC treated bacteria than in bacteria grown in iron enriched media in the majority of tested strains, which could explain the loss of drug activity in iron enriched media due to less drug uptake as the receptors are down regulated.

Another siderophore receptor (*fyuA*) exhibited GE that was strongly correlated with *fepA*. The expression of both *fepA* and *fyuA* was highest in CFDC treated bacteria, even higher than what was seen in iron depleted media without CFDC treatment, which means that the bacteria are actively expressing their siderophore receptors to acquire iron while being in an iron depleted environment. As CFDC was added to iron depleted media, it is expected that siderophores will be expressed more to acquire iron and their receptors will be expressed to assist in their uptake. As CFDC can chelate Fe^3+^, it can further starve the bacteria for iron and hence, can force greater expression of siderophore receptors to capture the siderophores loaded with iron.

In addition to siderophore receptors, bacteria can acquire iron using other receptors, such as *Klebsiella* ferric iron uptake (*kfu*), a regulator of iron transport responsible for acquisition of iron in *Klebsiella* species. Previous studies reported a link between *kfu* and ability of *K*. *pneumoniae* to cause invasive infections such as liver abscess, that was attributed to its role in mediating ferric iron uptake and modulating virulence and hypermucoviscosity. Besides, its presence in the clinical strains has been shown important for capsule formation and purulent tissue infections [40]. Ferric citrate transport is also one of the iron acquisition systems in bacteria. *FecA* usually binds to ferric citrate leading to conformational changes with subsequent transcriptional activation to uptake iron during iron starvation [41]. Overall, the expression of *fecA* was lower than the other receptors in this study, but it is still expressed by some strains particularly from I group. It was noticed that the presence of the *fecA* receptor alone, in addition to *fepA*, caused a significant increase in the MIC, and its presence with other receptors, mainly *kfu*, had a significant impact on the MICs as all the strains which were intermediately susceptible to the drug (MIC = 8 μg/ml) carried this gene. The expression of *kfu* was also associated with significant increase in CFDC MIC, when it was present alone with *fepA*, or with other receptors as *fecA* and/or *fyuA*. Correlation analysis showed a positive relation between CFDC MICs and GE of *fecA* receptor and also *kfu* receptor. Although the results were not statistically significant, probably due to small sample size, these results confirm our conclusion that these genes are linked to reduced susceptibility to CFDC as higher expression was associated with higher MICs, for example, a significantly higher expression of *kfu* was seen in R compared to S groups.

Overall, some strains demonstrated very unique GE patterns, suggesting dysregulation of iron uptake genes that can probably contribute to their altered susceptibility to CFDC. This is mainly true for R and I strains and some S strains. For example, one strain in the collection (KPN 8) had lower susceptibility to CFDC than the parent cephalosporines (CAZ and CPM). This was surprising, as all the other strains exhibited higher susceptibility to CFDC than the other cephalosporins and even meropenem, which was also reported by other investigators [42, 43]. We hypothesised that the cause of increased MIC of CFDC in this strain can be related to altered expression of iron acquisition genes. When this was tested, KPN 8 was found to carry three siderophore receptors (*fepA*, *fyuA*, and *iutA*) and did not harbour any iron transport genes (*fecA* and *kfu*). The expression of *fepA* receptor was low at the end of CFDC treatment (6 hrs). Our explanation to this phenomenon is that since there was very low expression of the receptor, CFDC was not able to penetrate the bacteria efficiently and therefore resulted in an increased MIC. This was supported by the finding that CFDC susceptible bacteria had higher expression of this gene, reflecting that this receptor is involved in the drug uptake. When KPN 8 was compared to another S strain (KPN 23), both of them had upregulated *fepA* and *fyuA* genes in iron enriched media, and therefore were able to maintain a non-resistant MIC in iron enriched conditions. Notably, KPN 23 had double MIC (8 μg/ml) compared to KPN 8 (4 μg/ml) in iron enriched media, while their MICs in iron depleted media was the same (2 μg/ml). As mentioned, KPN 8 devoid from iron uptake genes, while KPN 23 had *kfu* gene which was also overexpressed in all the growth conditions. In general, higher MIC in iron enriched media were found in strains expressing *kfu* and/or *fecA*, but it is important to consider other receptors that can be involved in iron uptake. Although the expression of *kfu* was very similar between KPN 23 (S) and KPN 68 (R), being consistent when these two strains were cultured in all the media, it is important to note that the increased susceptibility of KPN 23 can be due to the overexpression of *fepA* and the under expression of *fepA* is linked to reduced susceptibility in KPN 68, which exhibited unique patterns of expression for majority of receptors. This further signifies the importance of *fepA* receptor in the capturing of CFDC.

Considering other factors that might affect bacterial susceptibility to CFDC, it is important to mention that during the course of this study, some investigators reported the effect of mutations in the gene encoding colicin I receptor and catecholate siderophore receptor; *Cir* (also termed *CirA*), on susceptibility to CFDC in different species of GNB [33, 34, 44]. *CirA* is known as an OM receptor for linear Fe^3+^-enterobactin degradation products, as compared to *fepA* which is responsible for the uptake of intact Fe^3+^-enterobactin [45]; therefore, *fepA* receptor has high ligand affinity to Fe^3+^-enterobactin, while *cirA* is more specific to Fe^3+^- catecholates and their breakdown products containing Fe^3+^ [46]. For this reason, *fepA* was selected for testing in our study, and our results clearly showed its impact on drug entry to bacteria as CFDC MICs were negatively correlated with *fepA* expression.

It is worth mentioning that a very recent study by Lan et. al. in 2022 addressed the fact that susceptibility to CFDC in carbapenem-resistant *K*. *pneumoniae* can be affected by multiple factors, particularly *cirA* mutation and metallo-β-lactamases that can cause drug degradation. Using whole-genome sequencing, the latter study proved that high-level CFDC resistance could be mediated by the presence of NDM gene coupled with *cirA* mutation. They have shown that either factor present alone was not sufficient to cause CFDC resistance, while their combined effect jointly conferred high-level CFDC resistance [33]. Lan et. al. (2022) did not study the expression of iron transport receptors that might be involved in the drug entry, although the authors declared that *CirA* might not be the sole receptor for CFDC entry. Another study revealed that when only *cirA* was knocked out in *E*. *coli*, CFDC MIC was increased by 2-fold, whereas the MIC increased 16-fold by the double knockout of two receptors including both *cirA* and *fiu*; nevertheless, the bacteria remained susceptible [18]. These findings suggest that multiple iron transporters can contribute to the permeation of CFDC across the outer membrane; however, they are not the only factors affecting the drug activity. Importantly, none of the previous studies explored the gene expression of multiple iron transporters and their relation to the drug activity, as we did in our present study. Upregulation of *CirA*, in iron-limiting condition and downregulation in iron-rich condition was reported by other investigators [46]; thus, *CirA* GE was not tested in this study. We focused on testing siderophores, siderophore receptors and iron uptake genes which were not tested before; however, the effect of the *CirA* gene can be investigated in our bacterial collection in future studies using whole genome sequencing to find out mutations that can affect CFDC activity in our strains. As shown, the results reflect highly variable expression in between the different strains which was also seen in another study testing the expression of siderophores in *K*. *pneumoniae* strains [11]. Our findings strongly suggest that dysregulation of the expression of iron acquisition systems has an impact on susceptibility to CFDC, considering iron levels in the bacterial growth environment. Indeed, further investigations on more bacterial strains are needed to confirm these findings.

## Conclusion

When considering factors that can affect the activity of CFDC, iron seems to have a major impact as CFDC lost its activity when tested in iron enriched media and this could explain the increase in mortalities in patients receiving CFDC for the treatment of BSIs. Therefore, iron acquisition receptors can play a major role in the activity of CFDC. We identified enterobactin receptor (*fepA)* as a crucial receptor, mostly responsible for capturing of the drug and allowing its entry to the periplasm. The production of other iron uptake receptors (such as *fecA* and/or *kfu*) can reduce the requirement to express siderophores and hence, their uptake will be reduced. It is possible that when the bacteria harbour multiple iron acquisition systems, they do not rely on siderophores expression, and this could result in lowering the uptake of catechol-cephalosporin such as CFDC. Lastly, the variations and dysregulation in the GE of iron transport receptors in different growth conditions related to iron availability can contribute to the differences in the activity of CFDC. Alarmingly, one strain was highly resistant to CFDC, thus, the whole genome of this strain will be analysed through sequencing to identify causes of resistance. In addition to that, whole transcriptome sequencing is recommended in the future to provide a wholistic insight on different genes expressed during treatment with CFDC, for a better understanding of the diverse factors affecting the drug susceptibility in GNB with diverse characteristics. It will also help us to uncover underlying mechanisms of action that are not discovered and not understood about the drug.

## Supporting information

S1 Table List of the strains used in this study, their clinical sources with antibiotic susceptibility profiles. (XLSX)

S2 Table List of ATCC strains used in this study. (XLSX)

S3 Table Effect of iron on CFDC susceptibility in selected strains. (XLSX)

S4 Table Iron acquisition genes in all the strains investigated in this study. (XLSX)

## References

[1] Hamad M, Al-Marzooq F, Orive G, Al-Tel TH. Superbugs but no drugs: steps in averting a post-antibiotic era. Drug Discov Today. 2019;24: 2225–2228. doi: 10.1016/j.drudis.2019.08.004 31425765

[2] Daoud L, Al Marzooq F. PGN-015—Cefiderocol: A new weapon to fight antibiotic resistant *Klebsiella pneumoniae*. Int J Antimicrob Agents. 2021;58: 21003824. doi: 10.1016/j.ijantimicag.2021.106421.111

[3] Gareth M, Yoshinori Y, Keiko T, Masahiro K, Takuko S, Tsutae N. Antimicrobial resistance: Shionogi advocates policy change to address the public health threat. [cited 25 Jun 2022]. Available: https://www.nature.com/articles/d42473-020-00446-9

[4] Wu JY, Srinivas P, Pogue JM. Cefiderocol: A Novel Agent for the Management of Multidrug-Resistant Gram-Negative Organisms. Infect Dis Ther. 2020;9: 17–40. doi: 10.1007/s40121-020-00286-6 32072491 PMC7054475

[5] Sato T, Yamawaki K. Cefiderocol: Discovery, Chemistry, and In Vivo Profiles of a Novel Siderophore Cephalosporin. Clin Infect Dis. 2019;69: S538–S543. doi: 10.1093/cid/ciz826 31724047 PMC6853759

[6] Hennigar SR, McClung JP. Nutritional Immunity: Starving Pathogens of Trace Minerals. Am J Lifestyle Med. 2016;10: 170–173. doi: 10.1177/1559827616629117 30202269 PMC6124953

[7] Ouyang Z, Isaacson R. Identification and Characterization of a Novel ABC Iron Transport System, fit, in *Escherichia coli*. Infect Immun. 2006;74: 6949–6956. doi: 10.1128/IAI.00866-06 16982838 PMC1698097

[8] Meynard D, Babitt JL, Lin HY. The liver: conductor of systemic iron balance. Blood. 2014;123: 168–176. doi: 10.1182/blood-2013-06-427757 24200681 PMC3888285

[9] Holden VI, Wright MS, Houle S, Collingwood A, Dozois CM, Adams MD, et al. Iron Acquisition and Siderophore Release by Carbapenem-Resistant Sequence Type 258 *Klebsiella pneumoniae*. mSphere. 2018;3: e00125–18. doi: 10.1128/mSphere.00125-18 29669884 PMC5907654

[10] Cherayil BJ. The role of iron in the immune response to bacterial infection. Immunol Res. 2011;50: 1–9. doi: 10.1007/s12026-010-8199-1 21161695 PMC3085559

[11] Chen T, Dong G, Zhang S, Zhang X, Zhao Y, Cao J, et al. Effects of iron on the growth, biofilm formation and virulence of *Klebsiella pneumoniae* causing liver abscess. BMC Microbiol. 2020;20: 36. doi: 10.1186/s12866-020-01727-5 32070273 PMC7027070

[12] Bassetti M, Echols R, Matsunaga Y, Ariyasu M, Doi Y, Ferrer R, et al. Efficacy and safety of cefiderocol or best available therapy for the treatment of serious infections caused by carbapenem-resistant Gram-negative bacteria (CREDIBLE-CR): a randomised, open-label, multicentre, pathogen-focused, descriptive, phase 3 trial. Lancet Infect Dis. 2021;21: 226–240. doi: 10.1016/S1473-3099(20)30796-9 33058795

[13] Huang W-C, Wong M-Y, Wang S-H, Hashimoto M, Lin M-H, Lee M-F, et al. The Ferric Citrate Uptake System Encoded in a Novel blaCTX–M–3- and blaTEM–1-Harboring Conjugative Plasmid Contributes to the Virulence of *Escherichia coli*. Front Microbiol. 2021;12: 1226. doi: 10.3389/fmicb.2021.667782 34122381 PMC8187952

[14] Clinical and Laboratory Standards Institute. Performance Standards for Antimicrobial Susceptibility Testing. 30th ed. CLSI supplement M100. Clinical and Laboratory Standards Institute; 2020.

[15] Drieux L, Brossier F, Sougakoff W, Jarlier V. Phenotypic detection of extended-spectrum β-lactamase production in *Enterobacteriaceae*: review and bench guide. Clin Microbiol Infect. 2008;14: 90–103. doi: 10.1111/j.1469-0691.2007.01846.x 18154532

[16] Li J, Li C, Cai X, Shi J, Feng L, Tang K, et al. Performance of modified carbapenem inactivation method and inhibitor-based combined disk test in the detection and distinguishing of carbapenemase producing *Enterobacteriaceae*. Ann Transl Med. 2019;7: 566. doi: 10.21037/atm.2019.09.43 31807547 PMC6861796

[17] Rolston KVI, Gerges B, Shelburne S, Aitken SL, Raad I, Prince RA. Activity of Cefiderocol and Comparators against Isolates from Cancer Patients. Antimicrob Agents Chemother. 2020;64: e01955–19. doi: 10.1128/AAC.01955-19 32071053 PMC7179642

[18] Ito A, Sato T, Ota M, Takemura M, Nishikawa T, Toba S, et al. In Vitro Antibacterial Properties of Cefiderocol, a Novel Siderophore Cephalosporin, against Gram-Negative Bacteria. Antimicrob Agents Chemother. 2017;62: e01454–17. doi: 10.1128/AAC.01454-17 29061741 PMC5740388

[19] Hamad M, Al-Marzooq F, Srinivasulu V, Omar HA, Sulaiman A, Zaher DM, et al. Antibacterial Activity of Small Molecules Which Eradicate Methicillin-Resistant *Staphylococcus aureus* Persisters. Front Microbiol. 2022;13. doi: 10.3389/fmicb.2022.823394 35178043 PMC8846302

[20] Alshareef F. Protocol to Evaluate Antibacterial Activity MIC, FIC and Time Kill Method. Acta Sci Microbiol. 2021;4: 02–06. doi: 10.31080/ASMI.2021.04.0825

[21] Ribeiro-Júnior JC, Tamanini R, Alfieri AA, Beloti V. Effect of milk bactofugation on the counts and diversity of thermoduric bacteria. J Dairy Sci. 2020;103: 8782–8790. doi: 10.3168/jds.2020-18591 32828509

[22] Zhanel GG, Golden AR, Zelenitsky S, Wiebe K, Lawrence CK, Adam HJ, et al. Cefiderocol: A Siderophore Cephalosporin with Activity Against Carbapenem-Resistant and Multidrug-Resistant Gram-Negative Bacilli. Drugs. 2019;79: 271–289. doi: 10.1007/s40265-019-1055-2 30712199

[23] Ito A, Nishikawa T, Matsumoto S, Yoshizawa H, Sato T, Nakamura R, et al. Siderophore Cephalosporin Cefiderocol Utilizes Ferric Iron Transporter Systems for Antibacterial Activity against *Pseudomonas aeruginosa*. Antimicrob Agents Chemother. 2016;60: 7396–7401. doi: 10.1128/AAC.01405-16 27736756 PMC5119021

[24] Ibrahim M, Oyebanji E, Fowora M, Aiyeolemi A, Orabuchi C, Akinnawo B, et al. Extracts of endophytic fungi from leaves of selected Nigerian ethnomedicinal plants exhibited antioxidant activity. BMC Complement Med Ther. 2021;21: 98. doi: 10.1186/s12906-021-03269-3 33743702 PMC7981982

[25] Lawlor MS, O’Connor C, Miller VL. Yersiniabactin Is a Virulence Factor for *Klebsiella pneumoniae* during Pulmonary Infection. Infect Immun. 2007;75: 1463–1472. doi: 10.1128/IAI.00372-06 17220312 PMC1828572

[26] Rahmani HK, Tabar GH, Badouei MA, Khoramian B. Development of three multiplex-PCR assays for virulence profiling of different iron acquisition systems in *Escherichia coli*. Iran J Microbiol. 2020;12: 281–288. doi: 10.18502/ijm.v12i4.3930 32994898 PMC7502150

[27] Sanikhani R, Moeinirad M, Solgi H, Hadadi A, Shahcheraghi F, Badmasti F. The face of hypervirulent *Klebsiella pneumoniae* isolated from clinical samples of two Iranian teaching hospitals. Ann Clin Microbiol Antimicrob. 2021;20: 58. doi: 10.1186/s12941-021-00467-2 34465335 PMC8406009

[28] Yang M, Cousineau A, Liu X, Luo Y, Sun D, Li S, et al. Direct Metatranscriptome RNA-seq and Multiplex RT-PCR Amplicon Sequencing on Nanopore MinION–Promising Strategies for Multiplex Identification of Viable Pathogens in Food. Front Microbiol. 2020;11: 514. doi: 10.3389/fmicb.2020.00514 32328039 PMC7160302

[29] Karimaei S, Kalani BS, Shahrokhi N, Mashhadi R, Pourmand MR. Expression of type II toxin-antitoxin systems and ClpP protease of methicillin-resistant *Staphylococcus aureus* under thermal and oxidative stress conditions. Iran J Microbiol. 2021;13: 204–211. doi: 10.18502/ijm.v13i2.5982 34540156 PMC8408035

[30] Livak KJ, Schmittgen TD. Analysis of relative gene expression data using real-time quantitative PCR and the 2(-Delta Delta C(T)) Method. Methods San Diego Calif. 2001;25: 402–408. doi: 10.1006/meth.2001.1262 11846609

[31] Saderi H, Owlia P. Detection of Multidrug Resistant (MDR) and Extremely Drug Resistant (XDR) *P*. *Aeruginosa* Isolated from Patients in Tehran, Iran. Iran J Pathol. 2015;10: 265–271. 26351496 PMC4539747

[32] Lee YR, Yeo S. Cefiderocol, a New Siderophore Cephalosporin for the Treatment of Complicated Urinary Tract Infections Caused by Multidrug-Resistant Pathogens: Preclinical and Clinical Pharmacokinetics, Pharmacodynamics, Efficacy and Safety. Clin Drug Investig. 2020; 1–13. doi: 10.1007/s40261-020-00955-x 32700154 PMC7374078

[33] Lan P, Lu Y, Chen Z, Wu X, Hua X, Jiang Y, et al. Emergence of High-Level Cefiderocol Resistance in Carbapenem-Resistant *Klebsiella pneumoniae* from Bloodstream Infections in Patients with Hematologic Malignancies in China. Microbiol Spectr. 2022;0: e00084–22. doi: 10.1128/spectrum.00084-22 35323031 PMC9045219

[34] McElheny CL, Fowler EL, Iovleva A, Shields RK, Doi Y. In Vitro Evolution of Cefiderocol Resistance in an NDM-Producing *Klebsiella pneumoniae* Due to Functional Loss of CirA. Microbiol Spectr. 2021;9: e01779–21. doi: 10.1128/Spectrum.01779-21 34756080 PMC8579844

[35] Mohapatra SS, Dwibedy SK, Padhy I. Polymyxins, the last-resort antibiotics: Mode of action, resistance emergence, and potential solutions. J Biosci. 2021;46: 85. doi: 10.1007/s12038-021-00209-8 34475315 PMC8387214

[36] Page MGP. The Role of Iron and Siderophores in Infection, and the Development of Siderophore Antibiotics. Clin Infect Dis. 2019;69: S529–S537. doi: 10.1093/cid/ciz825 31724044 PMC6853763

[37] Wencewicz T, Miller M. Sideromycins as Pathogen-Targeted Antibiotics. Topics in Medicinal Chemistry. 2017. doi: 10.1007/7355_2017_19

[38] Jun J-B. *Klebsiella pneumoniae* Liver Abscess. Infect Chemother. 2018;50: 210–218. doi: 10.3947/ic.2018.50.3.210 30270580 PMC6167513

[39] El Fertas-Aissani R, Messai Y, Alouache S, Bakour R. Virulence profiles and antibiotic susceptibility patterns of *Klebsiella pneumoniae* strains isolated from different clinical specimens. Pathol Biol. 2013;61: 209–216. doi: 10.1016/j.patbio.2012.10.004 23218835

[40] Luo Y, Wang Y, Ye L, Yang J. Molecular epidemiology and virulence factors of pyogenic liver abscess causing *Klebsiella pneumoniae* in China. Clin Microbiol Infect. 2014;20: O818–O824. doi: 10.1111/1469-0691.12664 24804560

[41] Andrews SC, Robinson AK, Rodríguez-Quiñones F. Bacterial iron homeostasis. FEMS Microbiol Rev. 2003;27: 215–237. doi: 10.1016/S0168-6445(03)00055-X 12829269

[42] Stracquadanio S, Torti E, Longshaw C, Henriksen AS, Stefani S. In vitro activity of cefiderocol and comparators against isolates of Gram-negative pathogens from a range of infection sources: SIDERO-WT-2014–2018 studies in Italy. J Glob Antimicrob Resist. 2021;25: 390–398. doi: 10.1016/j.jgar.2021.04.019 34020073

[43] Falagas ME, Skalidis T, Vardakas KZ, Legakis NJ, on behalf of the Hellenic Cefiderocol Study Group. Activity of cefiderocol (S-649266) against carbapenem-resistant Gram-negative bacteria collected from inpatients in Greek hospitals. J Antimicrob Chemother. 2017;72: 1704–1708. doi: 10.1093/jac/dkx049 28369471

[44] Klein S, Boutin S, Kocer K, Fiedler MO, Störzinger D, Weigand MA, et al. Rapid Development of Cefiderocol Resistance in Carbapenem-resistant *Enterobacter cloacae* During Therapy Is Associated With Heterogeneous Mutations in the Catecholate Siderophore Receptor cirA. Clin Infect Dis. 2022;74: 905–908. doi: 10.1093/cid/ciab511 34079986 PMC8906715

[45] Miethke M, Marahiel MA. Siderophore-Based Iron Acquisition and Pathogen Control. Microbiol Mol Biol Rev. 2007;71: 413–451. doi: 10.1128/MMBR.00012-07 17804665 PMC2168645

[46] Gomes AÉI, Stuchi LP, Siqueira NMG, Henrique JB, Vicentini R, Ribeiro ML, et al. Selection and validation of reference genes for gene expression studies in *Klebsiella pneumoniae* using Reverse Transcription Quantitative real-time PCR. Sci Rep. 2018;8: 9001. doi: 10.1038/s41598-018-27420-2 29899556 PMC5998039

